# Use of PIXE/PIGE for sequential Ca and F measurements in root carious model

**DOI:** 10.1038/s41598-017-14041-4

**Published:** 2017-10-18

**Authors:** K. Yagi, H. Yamamoto, R. Uemura, Y. Matsuda, K. Okuyama, T. Ishimoto, T. Nakano, M. Hayashi

**Affiliations:** 1 Department of Restorative Dentistry and Endodontology, Osaka University Graduate School of Dentistry, Osaka, Japan; 2 Division of Clinical Cariology and Endodontology, Health Sciences University of Hokkaido Graduate School of Dentistry, Hokkaido, Japan; 3 Department of Dental Materials Sciences, Asahi University School of Dentistry, Gifu, Japan; 4 Division of Materials and Manufacturing Science, Osaka University Graduate School of Engineering, Osaka, Japan

## Abstract

The progress of caries has conventionally been evaluated by checking changes in mineral density using transverse microradiography (TMR). Recent advances have seen development of a new measurement system, using in-air micro proton induced X-ray/gamma-ray emission (PIXE/PIGE). PIXE/PIGE enables analysis of distributions and concentrations of multiple mineral elements in a carious lesion. The aim of this study was to evaluate the effectiveness of PIXE/PIGE for investigating the development of root caries. In summary, we successfully established a multi-elemental sequential measuring method using in-air micro-PIXE/PIGE to identify the dynamic distributions and concentrations of Ca and F in human root dentin. The PIXE/PIGE potentially offers a useful advantageous technique for studying carious development by using as a combination with conventional techniques such as TMR and Micro-computed tomography (µCT).

## Introduction

The onset and progress of caries have conventionally been investigated by measuring the changes in the mineral density of enamel and dentinal lesions using transmission micro radiography (TMR) as the effective standardized method. TMR evaluates demineralization and remineralization of carious lesions by depicting mineral densities in tooth substance^1–3^. Micro-computed tomography (µCT), which also has been frequently used in the measurement of the mineral densities in teeth, demonstrated the reasonable well correlation with TMR^4^. Results obtained by these TMR and µCT simply demonstrate the phenomena of the mineral changes in tooth substance, but do not explain the mechanisms of demineralization and remineralization of lesions at atomic levels.

In-air micro-particle induced x-ray and gamma ray emission (PIXE/PIGE) allows qualitative and quantitative analyses of Ca and F simultaneously by measuring the intensities of characteristic x-ray and gamma ray generated by proton bombardment to teeth^5–7^. Another major advantage of PIXE/PIGE is that it is a multi-elemental measuring method conducting in the atmosphere, and it does not require any pretreatment of specimens. This means that PIXE/PIGE allows serial measurements of Ca and F at different time points during demineralization and remineralization in carious lesions. In previous studies, PIXE/PIGE was used for analyzing the Ca and F, even Sr in sound and carious teeth^6,8–13^. However all these studies undertook a single measurement of the mineral components in tooth substance, or a measurement of single mineral elements in cavities. PIXE/PIGE has never effectively been used for analyzing the carious progress by measuring Ca changes. In the present study, we decided to prove the great potential of PIXE/PIGE to establish a sequential analyzing method of Ca and F in tooth structure. We used PIXE/PIGE with a precise superimposing technique to clarify demineralization mechanisms in root carious lesions.

Root caries is an important oral health issue especially in aging societies, which is the increasing fate of most developed countries. Elderly people, who have exposed root surfaces especially after periodontal treatments, or who have low saliva flow due to systemic medication, are susceptible to root carious lesions, since the critical pH of root surfaces that promotes demineralization is significantly higher than enamel^14,15^. Once a demineralized root surface develops a cavity, the carious lesions tend to expand and invade into proximal and sub-gingival areas along the enamel-cement junction although without severe pain^16^. Restoring such expanded root lesions is technically difficult because of poor accessibility and moisture control.

Therefore, prevention of root caries is vital for promoting life-long oral health. Fluoride has been proved to be an effective agent in preventing root caries, because it improves acid resistance and remineralization^17–19^. However the best way of using F to prevent root caries still needs to be explored.

The null hypothesis of this study was that in-air micro-PIXE/PIGE is ineffective in evaluating the distributions and concentrations of Ca and F in root dentin before and after demineralization. The validity of a sequential measurement of Ca and F using PIXE/PIGE was tested using a root carious model.

## Results

**Figure 1:**
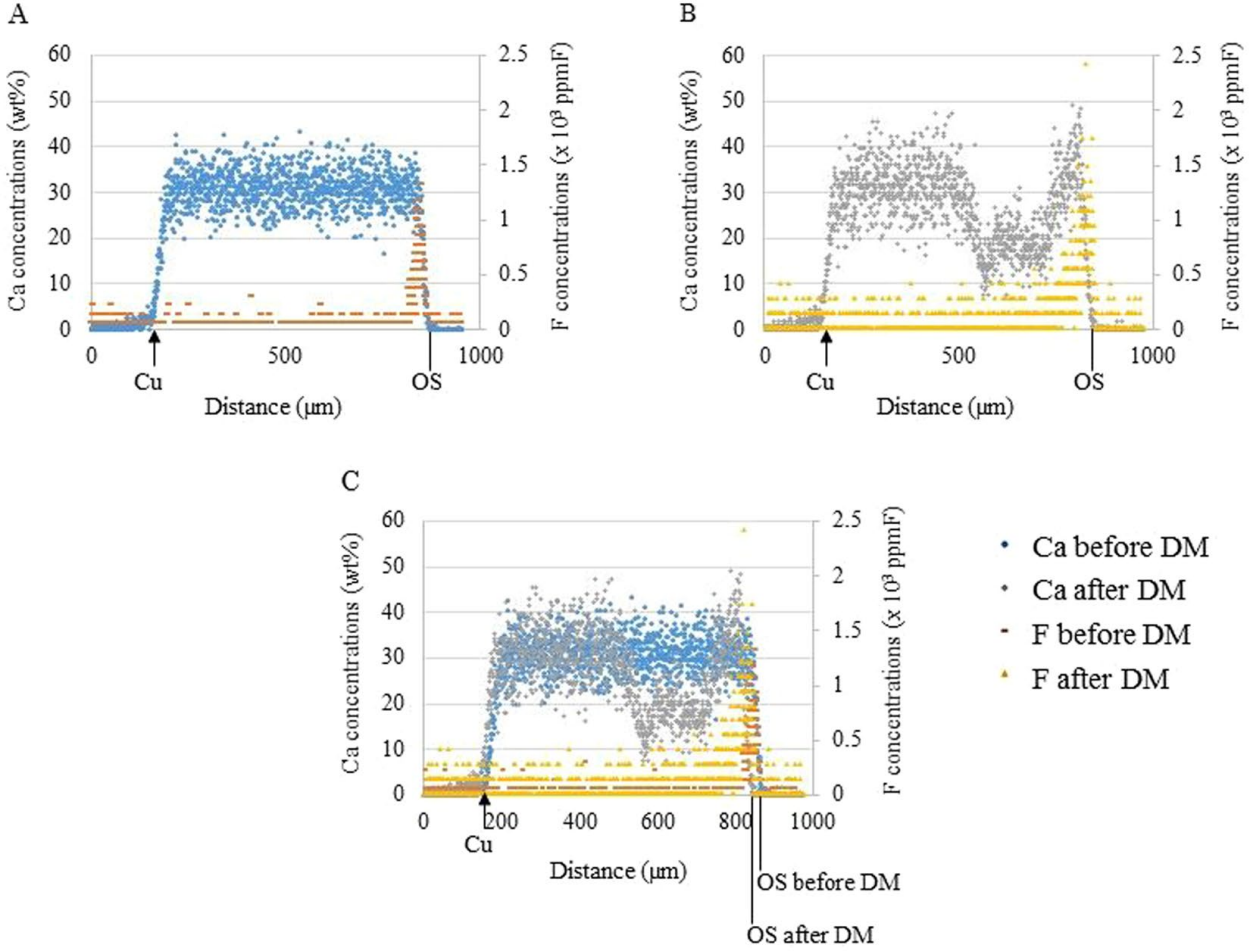
Distributions and concentrations of Ca and F before and after demineralization (DM). The results of Ca distributions obtained from line analysis using the same specimen before (**A**) and after (**B**) demineralization were superimposed on the basis of the reference point of the Cu foil (**C**).

The quantitative and qualitative measurements of Ca loss and F uptake in root dentin were established using PIXE/PIGE by superimposing the images obtained from line analyses of the same specimens before and after demineralization (Fig. 1).

**Figure 2:**
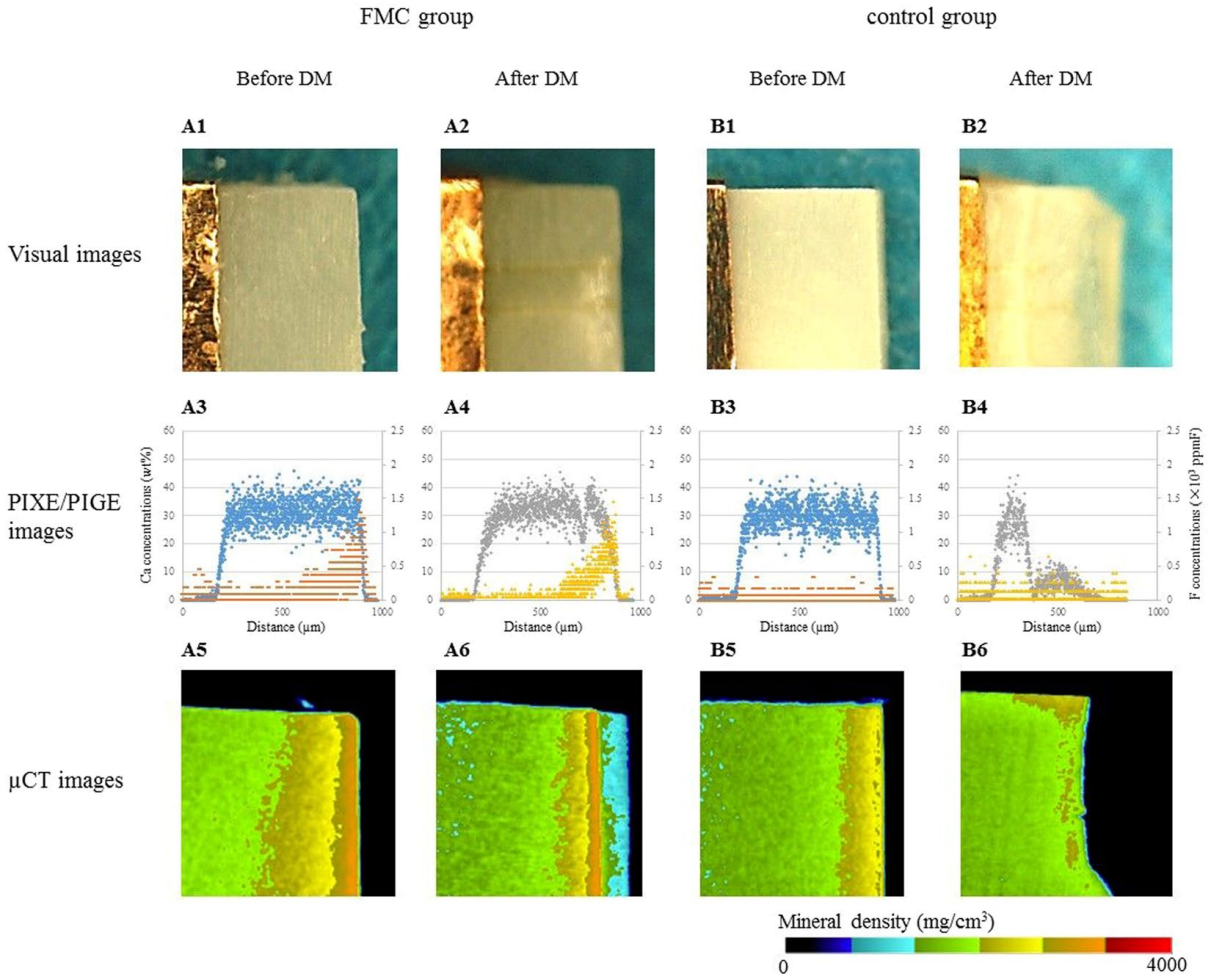
Images of FCM and control groups before and after demineralization (DM). Visual, the PIXE/PIGE and µCT images before and after DM in FCM group were presented as A1, 3, 5 and A2, 4, 6, respectively. Those in control group were B1, 3, 5 and B2, 4, 6. Symbols in the PIXE/PIGE images are identical to those in Fig. 2. The mineral density in µCT images was color-coded in mg/cm^3^.

Figure 2 shows representative visual, PIXE/PIGE and µCT images of root dentin in the FCM and control groups before and after demineralization. According to the findings of both PIXE/PIGE and µCT images, the root dentin in the FCM group (Fig. 2-A4 and A6) retained larger amounts of Ca after demineralization compared to the control (Fig. 2-B4 and B6). The PIXE/PIGE images showed a substantial amount of F uptake in the FCM group compared to that of the control (Figs 2-A3,4 and B3, 4). The mineral densities demonstrated by the µCT images agreed with the Ca concentrations in the PIXE/PIGE.

**Figure 4:**
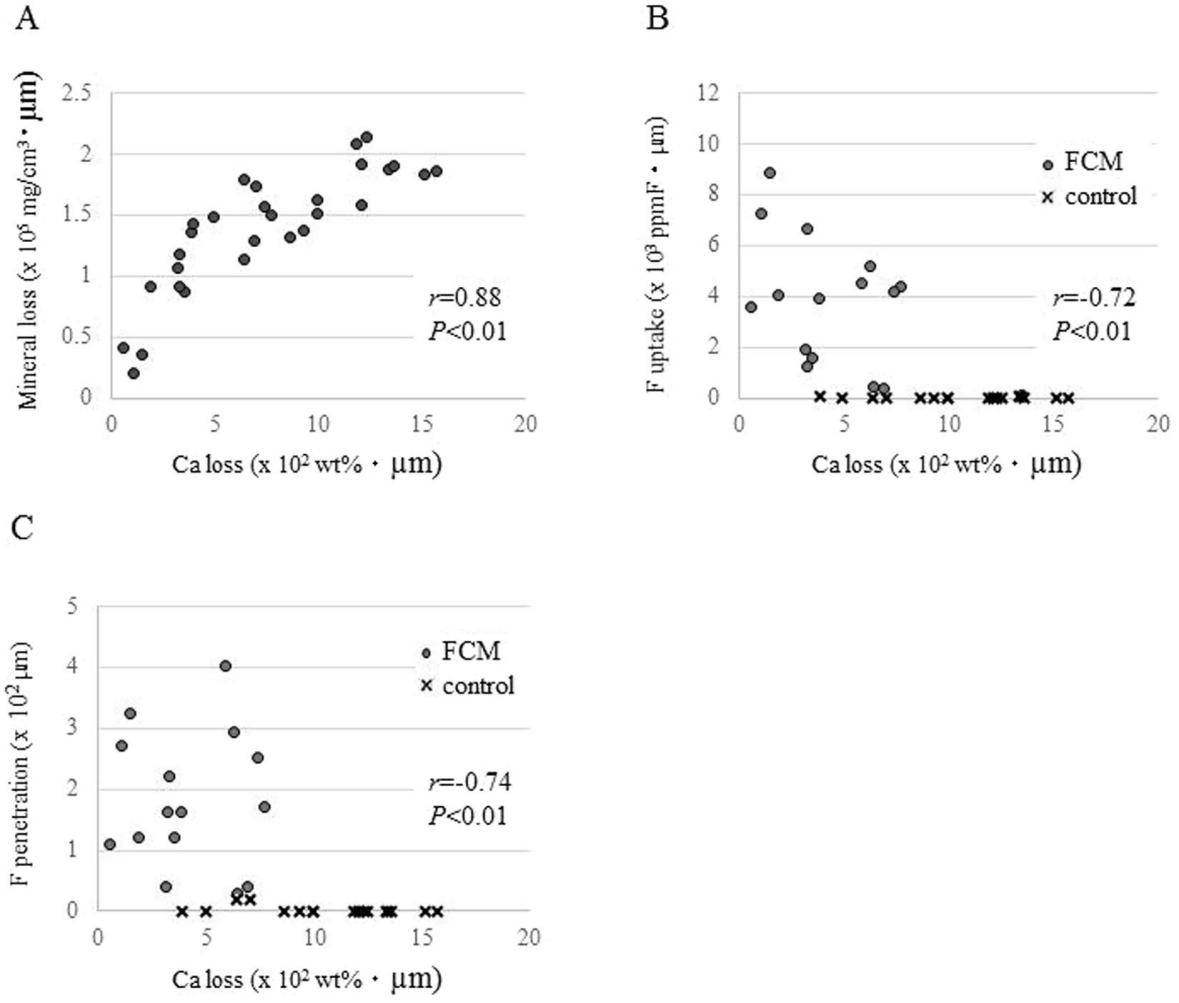
Correlations between the Ca loss and the mineral loss, the F uptake and the F penetration. (**A**) The correlation between the Ca loss measured by PIXE/PIGE and the mineral loss by µCT. (**B**) The correlation between the F uptake before demineralization and the Ca loss. (**C**) The correlation between the F penetration before demineralization and the Ca loss.

**Figure 3:**
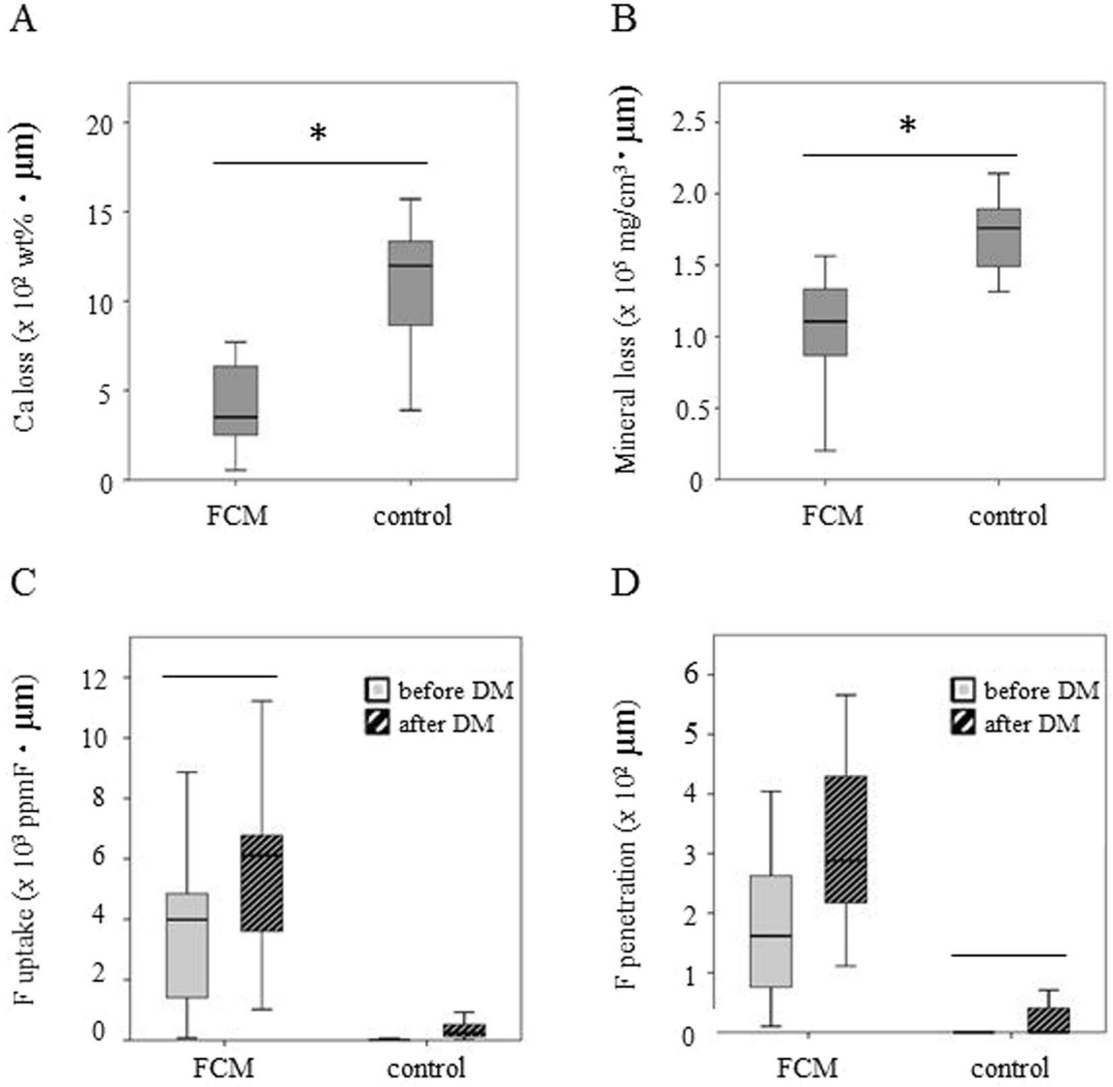
The Ca loss, the mineral loss, the F uptake and the F penetration before and after demineralization (DM). (**A**) Ca loss after DM measured by PIXE/PIGE. (**B**) Mineral loss after DM measured by μCT. (**C**) F uptake before and after DM. (**D**) F penetration before and after DM. n = 9, Mann-Whitney *U*-test, *P < 0.05, −: Groups connected with lines showed no significant differences.

The Ca loss in the FCM group was 5.4 × 10~7.7 × 10^2^ wt% · µm (median: 3.5 × 10^2^ wt% · µm); while that in the control group varied from 3.9 × 10^2^~1.6 × 10^3^ wt% · µm (median: 1.2 × 10^3^ wt% · µm) (Fig. 3A). The FCM group showed a significantly smaller loss of Ca than the control group (Mann-Whitney *U*-test, *P* < 0.05). The same phenomenon was observed by the mineral loss detected by µCT. The mineral loss in the FCM group was 2.0 × 10^4^~1.6 × 10^5^ mg/cm^3^ · µm (median: 1.1 × 10^5^ mg/cm^3^ · µm); while that in the control group was 1.3 × 10^5^~2.1 × 10^5^ mg/cm^3^ · µm (median: 1.8 × 10^5^ mg/cm^3^ · µm) (Fig. 3B).

The F uptakes before and after demineralization in the FCM group were 7.2 × 10~8.9 × 10^3^ ppmF · µm (median: 4.0 × 10^3^ ppmF · µm) and 1.0 × 10^3^~1.1 × 10^4^ ppmF · µm (median: 6.0 × 10^3^ ppmF · µm); for those in the control group, they were 0.0~6.8 × 10 ppmF · µm (median: 1.0 × 10 ppmF · µm) and 8.1~1.2 × 10^3^ ppmF · µm (median: 2.0 × 10^2^ ppmF · µm) (Fig. 3C). The FCM group showed significantly higher F concentrations than the control group both before and after demineralization (Mann-Whitney *U*-test, *P* < 0.05).

The F penetrations before and after demineralization in the FCM group were 1.0 × 10~4.0 × 10^2^ µm (median: 1.6 × 10^2^ µm) and 1.1 × 10^2^~5.7 × 10^2^ µm (median: 2.9 × 10^2^ µm); for those in the control group, they were 0.0~2.0 × 10 µm (median: 0.0 µm) and 0.0~7.0 × 10 µm (median: 0.0 µm) (Fig. 3D). The FCM group showed significantly greater F penetration than the control group both before and after demineralization (Mann-Whitney *U*-test, *P* < 0.05).

The Ca loss measured by PIXE/PIGE and the mineral loss by µCT showed a significant positive correlation (Spearman’s rank correlation, *r* = 0.88, *P* < 0.01) (Fig. 4A). The F uptake and the Ca loss after demineralization showed a significant negative correlation (Spearman’s rank correlation coefficient, *r* = −0.72, *P* < 0.01) (Fig. 4B). The F penetration also showed a negative correlation to the Ca loss (Spearman’s rank correlation coefficient, *r* = −0.74, *P* < 0.01) (Fig. 4C).

## Discussion

A novel finding of the present study was that by using in-air micro-PIXE/PIGE we successfully established a sequential measuring method to identify the distributions and concentrations of Ca and F in the human root carious model. The notably superior characteristic of PIXE/PIGE over the conventional TMR or µCT is the ability to measure the distributions and concentrations of Ca and F simultaneously using the combination of PIXE and PIGE: such multi-elemental analyses also provided better understanding the mechanisms of carious progression and remineralization at the atomic levels. In addition, the PIXE/PIGE offers simpler and easier experimental procedures compared with TMR. There were no cracks or shrinkage of the specimens during subsequent sequential observations, because the analyses were conducted in air without damage by pretreatments. Superimpose the mappings of Ca and F taken from identical specimens before and after demineralization was possible using the mathematical software and the Cu reference point. This technique allows authentic sequential comparison of tooth components and was effective in assessing carious progress.

Electron probe microanalysis (EPMA) and energy dispersive X-ray spectrometry (EDS) are another options to depict the distributions of major comportments of tooth substance such as Ca, F, Sr and P^20–26^. However, sequential analysis using EPMA is not possible, since again it requires destructive pretreatment such as drying or evaporating treatment of a specimen. EDS also needs destructive pretreatment of a specimen for the observation using scanning electron microscope.

The sample size in the present study was set with reference to other previous studies^3,17,27^, which demineralization and remineralization of dentin could be appropriately evaluated. In addition, the samples in FMC and control groups were taken from identical teeth as pairs in order to minimize individual differences.

A beam spot of the PIXE/PIGE as small as 1μm enabled a high-resolution analysis of the distributions of each mineral element in dentin. The validity of the new system was proved by the high positive correlation between the Ca loss and the mineral loss measured by the µCT (*r* = 0.88, Fig. 4A). Areas with low mineral densities confirmed by the µCT had limited possibility of retaining other mineral components such as Sr and P after demineralization. Taking all these benefits and advantages together, in-air micro PIXE/PIGE using as a combination with conventional TMR or µCT offers more detailed information on carious lesion.

A negative correlation between the F uptake from the FCM and Ca loss was confirmed (Fig. 4B). Similar results, showing the preventive effects of F on the carious onset and progress in enamel and dentin, were demonstrated in previous studies^17,28–30^. FCMs release a substantial amount of F in acidic conditions rather than in neutral conditions, since F, as anion, is easily eluted in acidic conditions producing a high concentration of hydrogen cation. Released F may be absorbed into enamel and dentin through the surface because of its small ionic radius^10^. In the present study, there was no significant difference in the F uptake before and after demineralization, but the F penetration after demineralization was greater than that before in the FCM group (Fig. 3C and D). These results suggested that a considerable amount of F was able to penetrate into deeper parts of the dentin through its porous structure after demineralization.

The F penetration into tooth structure from the FCMs showed two types of F to tooth interactions: incorporation into the crystal lattice; and adsorption into the tooth structure. Since F incorporated in the crystal lattice is chemically stable^31,32^, such F could contribute to the acid resistance of dentin by producing a dense structure. On the other hand, F absorbed to the tooth surface also elutes from the tooth when tooth mineral is dissolved in a demineralizing solution. This suggested that F absorbed by the tooth could be a source of remineralization, and thus protective against demineralization^32,33^. However, it is still unclear the most effective way of applying F to tooth interaction and the best concentrations and distributions in enamel and dentin necessary to prevent mineral loss.

In the present study, there were negative correlations between the accumulated amount and penetrated depth of F and Ca loss before demineralization (Fig. 4B and C). However, there was no significant correlation between the F penetration and Ca loss, when the data of FCM alone without the control were analyzed (r = 0.18, *P* > 0.05, Fig. 4C). This may be because the surface density of F is critical rather than its penetration for carious prevention of dentin. In previous studies, which investigated the F uptake in a cavity by PIXE, the distributions and concentrations of F from FCMs to enamel and dentin showed different patterns, depending on the testing conditions such as pH, the F concentrations and the targeted tooth substances^34–36^. For future studies, the PIXE/PIGE measurement should be controlled as an effective method to clarify the detailed reaction of F and other atoms with tooth substance with the aim of developing effective materials and methods for prevention of caries.

In summary, we successfully established a multi-elemental sequential measuring method using in-air micro-PIXE/PIGE system to identify the distributions and concentrations of Ca and F in the human root carious models. The PIXE/PIGE potentially offers a useful advantageous technique for studying carious development by using as a combination with conventional techniques such as TMR and µCT.

## Methods

All experiments are carried out in accordance with protocols approved by the Research Ethics Committee of Osaka University Graduate School of Dentistry (H25-E28). Teeth were collected after written patient informed consent, under protocols approved by the research ethics committee.

### Specimen Preparations

A total of nine third molars free from caries, that had been extracted for orthodontic and periodontal reasons at Osaka University Dental Hospital were used as materials. They were stored in 100% humidity for periods of shorter than 6 months after extraction.

**Figure 5:**
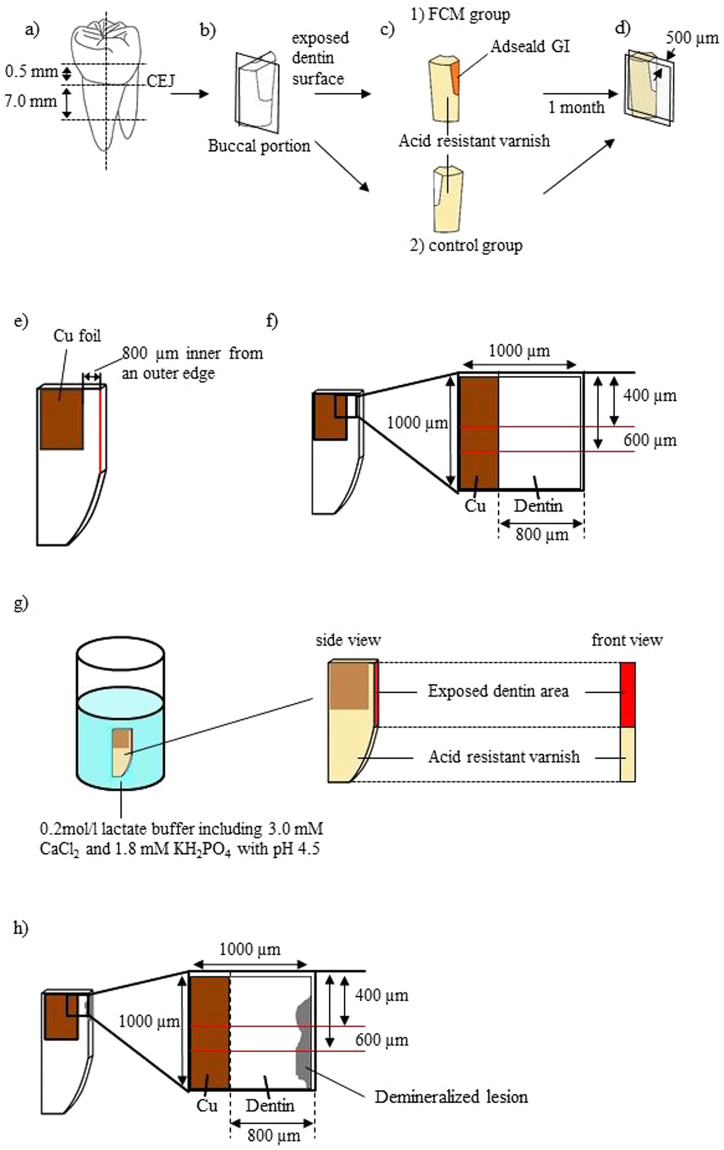
Preparation of root dentin specimens for Ca and F measurements using PIXE/PIGE. (**a**) Each tooth was sectioned perpendicularly to its axis. A buccal half was used for the experiments. (**b**) The buccal surface of the root block was cut until dentin was exposed, and the root block was sectioned in two longitudinally at the center of the exposed dentin surface. (**c**) The exposed root dentin area of the mesial block was covered with a FCM (c-1: FCM group), and that of the distal block served as a control without FCM (c-2: control group). Each specimen was coated by an acid resistant varnish leaving the exposed dentin area. The specimens were immersed in saline for 1 month at 37 °C. (**d**) The aforementioned acid resistant varnish and the FCM were removed and each dentin block was sliced parallel to the longitudinal axis and perpendicularly to the exposed root dentin area to make a 500 µm section for the Ca and F measurements. (**e**) A Cu foil was placed on the cutting plane of specimen at 800 µm inner from an outer edge of the exposed root dentin. (**f**) The first PIXE/PIGE measurement was performed before demineralization. (**g**) Demineralization of dentin: immersed in 10 ml of demineralization solution for 3 days. (**h**) The second PIXE/PIGE measurement was performed after demineralization.

The preparations of specimens were as shown in Fig. 5. Each tooth was sectioned perpendicularly to the axis at the points of 0.5 mm upper and 7.0 mm below from cement-enamel junction (CEJ) using a low-speed saw (IsoMet, Buehler, Lake Bluff, IL, USA) with a diamond disc (15LC Diamond Wafering Blade, Buehler) under deionized running water (Fig. 5a). Then, the buccal half of the root block was used for the experiment (Fig. 5b). The buccal surface of the root block was cut until dentin was exposed. Then, the root block was sectioned in two longitudinally at the center of the exposed dentin surface (Fig. 5b). The exposed root dentin area of the mesial section was covered with a fluoride-containing material (FCM) (Adseald^®^GI, Kuraray Noritake Dental, Tokyo, Japan), and the distal section served as a control without FCM. Each specimen was coated with an acid resistant varnish, leaving the exposed dentin area, in order to allow fluoride uptake only from the exposed dentin (Fig. 5c). Finally, each specimen was immersed in isotonic sodium chloride solution (saline) for one month at 37 °C. The solution was changed every week.

After one month, the aforementioned acid resistant varnish and the FCM were removed by hand instruments under observation by a microscope. Each dentin block was sliced parallel to the longitudinal axis and perpendicularly to the exposed root dentin area to make a 500 µm section for the Ca and F measurements (Fig. 5d). A copper (Cu) foil with a thickness of 4 µm was placed on the cutting plane of each specimen in order to make a reference point for comparing the Ca concentrations and F uptake before and after demineralization (Fig. 5e). The Cu foil was set at 800 µm inside from an outer edge of the exposed root dentin.

### Measurement of Ca and F Distributions and Concentrations before Demineralization

The Ca and F distributions and concentrations in specimens were measured using the PIXE/PIGE system at the Wakasa Wan Energy Research Center according to the previous report 6. A 2.5-MeV proton beam was used to bombard specimens in the air. The beam spot size was less than 10 µm, with a beam current of approximately 50 pA. The nuclear reaction^9^F (ρ,αγ)^16^ O was used to measure the F content. The gamma-rays in this reaction were detected using a 3”BGO detector, located just behind the sample stage. Ca concentration was measured by PIXE. X-rays were simultaneously detected with a pure germanium detector without an X-ray absorber, which was placed at an angle of 130° with respect to beam axis, in a vacuum. The beam current was monitored by counting silicon K X-rays passing a silicon nitride window using a Si-PIN X-ray detector. Quantitative results were obtained by calibrating the PIGE yield using hydroxyl apatite (HA) with varying levels of fluoridation as reference materials (Ca_10_(PO_4_)_6_(OH)_2−2x_F_2x_ with x = 0, 0.25, 0.5, 0.75, 1; HOYA Technosurgical Co., Ltd.).

At the measurement of Ca and F distributions and concentrations, an area of 1000 × 1000 µm^2^ of the measuring surface, including the Cu foil was scanned on a sample stage in the air (Fig. 5f). For each specimen, two lines at positions of 400 and 600 µm from the top edge in the scanning area were selected and subsequently analyzed.

### Demineralizing Treatment

After the measurement of Ca and F, the surface of each specimen, except for the exposed root dentin area, was covered again with the acid resistant varnish to allow demineralization only from the exposed root dentin. Each specimen was immersed in 10 ml of demineralizing solution for 3 days at 37 °C to simulate carious attack (Fig. 5g). The components of the demineralizing solution were 0.2 mol/l lactate buffer including 3.0 mM CaCl_2_ and 1.8 mM KH_2_PO_4_ with pH 4.5^37^. After three days, the exposed dentin area was washed with deionized water for 30 seconds and the acid resistant varnish was removed from the specimens. The specimen was stored in 100% humidity until the second PIXE/PIGE measurement.

### Measurement of Ca and F Distributions and Concentrations after Demineralization

After demineralization, the Ca and F distributions and concentrations in the identical specimens were measured again at the same points of before demineralization measured using the PIXE/PIGE (Fig. 5h).

### Comparisons of Ca and F before and after Demineralization

Distributions and concentrations of Ca and F before and after demineralization were converted from calibration curves obtained from reference materials using the software (Tooth linescan analyzer; Wakasa Wan Energy Research Center). The outermost surface of the lesion was defined as the position containing 5% of the average Ca of intact dentin; the innermost surface of the demineralized lesion was defined as the position containing 95% of the average Ca of intact dentin.

In analyzing the demineralization of the root dentin, the results of Ca distributions obtained from the line analysis using the same specimen before (Fig. 1A) and after (Fig. 1B) demineralization were superimposed on the basis of the reference point of the Cu foil (Fig. 1C). On the superimposed image, the average Ca and F concentrations from the outermost surface to the Cu reference point were calculated at 10 µm intervals. The Ca loss (wt% · µm) was calculated by integrating the difference of average Ca densities before and after demineralization. The F uptake (ppmF · µm) before and after demineralization was calculated by cumulating the average F densities. The F penetration depth was taken as a distance from the outermost surface to the point showing undetectable F concentration.

### Micro-CT Scanning before and after Demineralization

Before and after demineralization, all specimens were scanned by µCT (inspeXio SMX-100CT; Shimadzu, Kyoto, Japan). The specimens were scanned with a spatial resolution of 8.1 µm and their projection images were collected at 40 kV and 100 µA using 180° rotation with 0.6° per projection step. An aluminum filter with thickness of 0.1 mm was placed in the beam path to remove low-energy radiation. Data were acquired with 512 × 512 pixel resolution and 8.1 µm isotropic voxel sizes. For calibrating mineral density, a series of reference phantoms were also scanned, which included four hydroxyapatite disks with different concentrations (100, 200, 300, 400 mg/cm^3^) and an aluminum pole (1550 mg/cm^3^).

Two-dimensional images of the specimens were reconstructed using a CT-analyzer software (CT-solver; Shimadzu, Kyoto, Japan). The stacked images were analyzed with Image J software (NIH, Bethesda, MD, USA) to produce an overall mineral profile of the two lines, which were the identical locations as scanned by the PIXE/PIGE system. Mineral loss (mg/cm^3^ · µm) was calculated by integrating the difference of the mineral content profiles of the identical specimens before and after demineralization.

### Statistical Analyses

Statistical analyses were performed with IBM SPSS Statistics 22 (IBM, Armonk, New York). The Ca loss, the F uptake, and the mineral loss were compared between FCM and control groups by Mann-Whitney U-test at a 95% level of confidence. The correlations between the Ca loss detected by PIXE and the F uptake, F penetration, and the mineral loss by µCT were evaluated by Spearman’s rank correlation coefficient at a 99% level of confidence.
